# Development of Quality Indicators for Geriatric Home Enteral Nutrition (HEN) Services

**DOI:** 10.3390/nu15143119

**Published:** 2023-07-12

**Authors:** Nikolina Jukic Peladic, Paolo Orlandoni

**Affiliations:** 1 Vivisol Srl., Clinical Nutrition Unit, National Institute of Health and Science on Aging, IRCCS INRCA Ancona, Via della Montagnola 81, 60127 Ancona, Italy; 2 Clinical Nutrition Unit, National Institute of Health and Science on Aging, IRCCS INRCA Ancona, Via della Montagnola 81, 60127 Ancona, Italy; p.orlandoni@inrca.it

**Keywords:** Home Enteral Nutrition (HEN), quality improvement, quality assessment, indicators, geriatric patients

## Abstract

The evidence on the safety, efficacy and patient centeredness of Home Enteral Nutrition (HEN) services is scarce. In 2015, we carried out a search of the literature to identify specific indicators for HEN services as tools to be used to assess the quality of INRCA HEN services. No specific indicators for HEN services were found. Through a subsequent search of the literature, we have identified the appropriate methodology to define quality indicators and developed eight (8) specific indicators to track the quality of our HEN service for geriatric patients. Following Donabiedan’s classification, we have defined two structure indicators, two process indicators and four outcome indicators that are presented in this manuscript. Though they may be used to make a comparison of HEN services for geriatric patients and to monitor the quality of therapy provided at patients’ homes, the definition of quality system indicators for HEN services requires the additional joint efforts of experts in the field of nutrition and the scientific community for their validation.

## 1. Introduction

When a patient’s condition is stable, the home environment is suitable, and when the patient and/or caregiver have been trained sufficiently to perform the tasks associated with Enteral Nutrition (EN) at home, EN therapy, or tube feeding, may be provided at a patient’s home. The administration of Enteral Nutrition at home—Home Enteral Nutrition (HEN)—allows the early discharge of patients from hospitals with the consequent saving of costs related to hospitalization, and it positively affects their quality of life, given that patients normally prefer the family environment rather than the hospital [1,2].

Numerous dedicated services for Home Enteral Nutrition (HEN) have arisen worldwide, to the point that HEN nowadays is the most widespread home-administered therapy [3,4]. While the protocols and programs of different HEN services have been described to some extent in the literature, the evidence on their quality is still very scarce [5,6,7,8]. The quality of any healthcare service is given by its ability to be patient centered, effective and to guarantee patient safety [9,10,11]. To measure the quality of healthcare and its improvement, quality indicators are normally used [12,13,14]. Indicators are quantitative or qualitative variables, which allows for a synthetic evaluation of complex phenomena, such as that of quality. They may be used for evaluation of results achieved over time, for comparison of results of different services (benchmarking) and for gap analysis [15,16]. Gap analysis, in particular, allows for measuring how far the healthcare provided is from the standards defined by the scientific evidence as the best practices relative to specific pathologies, patient groups and treatments. Though the diagnostic or treatment processes defined as standards are ideally derived from the best scientific evidence, sometimes high quality scientific evidence are not available and expert consensus, laws and regulations, or even internal protocols, may be used to define the aims that a health organization or service should pursue [17,18]. Quality indicators should also be used to evaluate the appropriateness of the methods used to provide the HEN. Unfortunately, indicators developed specifically to evaluate HEN services are not available [19]. One of the reasons is that, until very recently, the guidelines on HEN, which are the basis for development of indicators, were not available [20,21]. Once available, the guidelines mostly simply recommended certain behaviors without defining precise and measurable quality standards.

Actually, there are some indicators in the literature that are useful in evaluating the quality of the nutritional therapy itself [22,23]. The quality indicators for home EN therapy should measure, in the first place, to what extent EN therapy has been provided at home and with what level of safety and effectiveness it is carried out in that setting. To be efficient, home therapy should ensure that most of the therapy, as well as the prevention and management of its complications, can be resolved at home, without the need to return to hospital, either in the form of an outpatient visit or hospitalization. This aspect assumes even greater importance for older patients for whom transportation is particularly difficult and hospital visits often lead to a worsening of their clinical conditions [24]. It should also be considered that HEN therapy is provided in coproduction between the health staff and patients or their caregivers. Consequently, both the characteristics and actions of the health staff, and those of caregivers and patients who are trained to perform some important tasks at home, may affect the outcomes [25,26,27,28,29].

The center for Clinical Nutrition Unit at the geriatric research hospital INRCA, Ancona, has provided HEN to geriatric patients (mean age 84.2 ± 9.6 years, Geriatric Nutritional Risk Index (GNRI) 81.4 ± 1.1, 85% with multiple diseases) since 2005. In this manuscript, we present eight indicators that we developed after our search of the literature carried out in 2015 did not allow for the identification of the specific quality indicators for HEN services already available. The indicators presented in this manuscript allow for the assessment of the existence of the prerequisites for an efficient transfer of therapy at home (structural and process indicators), and the outcomes of a therapy provided in that setting. They could be used to compare the procedures and results of the different HEN services and to identify the best practices. We also present the values of eight indicators that have been tested at INRCA HEN service since 2015.

## 2. Materials and Methods

### 2.1. INRCA HEN Service

All details about the characteristics of HEN service provided by Clinical Nutrition Unit of INRCA Ancona have been previously published [30]. A multi-professional team formed by physicians specializing in clinical nutrition, as well as nurses and dietitians, operates partly in hospital and partly at patients’ homes. Caregivers and patients are trained to administer the therapy at home and to deal with possible difficulties and complications by contacting the right interlocutor from a multi-professional team. This following data, which are indispensable for the elaboration of indicators, are systematically gathered and updated for each patient: demographic data (gender, date of birth, living conditions), clinical data (diseases, drug therapies, pressure ulcers), nutrition-related data (Body Mass Index (BMI), albumin and prealbumin values, reasons for HEN, administration rout, nutrition therapy), results of scheduled examinations and assessments performed during home visits, information on tube-related, gastrointestinal and metabolic complications, information on outpatient hospital visits and hospitalizations during the current month, length of hospital stay, diagnoses at hospital discharge, frequency of programmed hospital visits for nutritional assessment and changes of the nutritional therapy and causes of death. All data are registered on a dedicated internet database—Vivimedical—within case report schedules and are accessible only to home and hospital staff of INRCA HEN service, in real time.

### 2.2. INRCA HEN Service Indicators

A search of the literature was performed from January to May 2015 with the aim of identifying appropriate indicators to assess the quality of INRCA HEN service. The search was conducted in Pubmed, Medline, EMBASE and in the gray literature. Backward citation searching was conducted by inspecting the reference list of the literature found. Search terms included: healthcare indicators, quality indicators, home care, nutritional therapy, enteral therapy, home nutritional and enteral therapy, HEN guidelines, HEN standards, HEN regulations, geriatrics, older adults and combinations of these. The search was limited to English language and Italian language manuscripts and documents. The searches were conducted by one researcher (N.J.P.) and checked by another researcher (P.O.). The inclusion criteria were: studies providing the evidence on the quality of Home Enteral Nutrition therapy, older subjects and quality indicators. The exclusion criteria were: patients on oral nutrition supplements, studies involving children and young adults, studies with abstract only. Our search did not result in the identification of specific indicators for HEN. Thus, in the period from June to October 2015, we carried out a search of the literature to identify the documents containing the methodology for the development of quality indicators for our HEN service. The search was conducted in Pubmed, Medline, EMBASE and in gray the literature. Backward citation searching was conducted by inspecting the reference list of the literature found. The terms used to search for documents and studies, exclusively in English and Italian language, were: methods for defining quality indicators, construction of health quality indicators, types of health quality indicators [31,32]. Studies and documents assessing the quality by means and methods different than indicators were not considered. The searches were conducted by one researcher (N.J.P.) and checked by another researcher (P.O.). At the term of the search, documents were analyzed and, in the same year, we developed eight indicators to measure the safety and efficacy of INRCA HEN service for geriatric patients, following the Donabiedian classification [33]. Indicators presented in this manuscript were updated in 2020, after the publication of European Society for Parenteral and Enteral Nutrition (ESPEN) guidelines, to assess compliance of our service with the most up-to-date standards. Structural indicators—Correspondence of structures responsible for provision of HEN to accreditation standards and Adequacy of places and methods of preserving enteral feeding tube formulas and devices at patient’s home—measure the compliance of the IRCCS INRCA HEN service with accreditation standards established by the Italian Ministry of Health guidelines. Process indicators—Frequency of follow-up visits at patient’s home and Frequency of outpatient visits for patients treated with HEN—were elaborated in order to track the operational process and measure its compliance with available reference standards defined by the ESPEN guidelines, clinical pathways and internal procedures and protocols [20,34,35]. Outcome indicators—Frequency of complications of HEN therapy, Frequency of hospitalizations for complications of HEN therapy, Length of hospital stays for complications of HEN therapy and Frequency of deaths following hospitalizations for complications of HEN therapy—document changes in intermediate and final clinical outcomes of patients.

## 3. Results

Eight indicators presented in the following text and tables—two structural, two process and four outcome—were developed for the purposes of quality assessment of INRCA HEN service for geriatric patients. For each indicator, we provide the explanation on its usefulness—the “why measure it?” section—the information about the available reference standards, how to measure the indicator, and the results registered by INRCA Clinical Nutrition Unit in the period 2015–2021, measured by each indicator. Values of some indicators are not available for the year 2020 given that the performance of the service was affected by the COVID-19 pandemic.

1. Structural indicator: Correspondence of structures responsible for provision of HEN to accreditation standards

Why measure it? In order to ensure that therapy is delivered safely and efficiently at home, the Italian Ministry of Health, in 2006, adopted guidelines which define the characteristics that organizational structures and multi-professional teams must have in order to be accredited as centers for HEN [20]. Nevertheless, very few regions, which are responsible for enforcing national directives on their territory, have defined the essential characteristics of structures of HEN centers by their regulations, so that the existing HEN services in Italy are very heterogeneous with respect to their structural, technological and organizational characteristics. Compliance with requirements for accreditation defined by Italian national guidelines is the first and essential condition that has to be met in order to guarantee patient’s safety. Information provided by this indicator is useful in assessing the patient’s safety and to compare the costs (expenditure indicators) and the outcomes of different centers (benchmarking). Recommendations about staff composition are also given in ESPEN guidelines (R 53) [21].

Standard: Guidelines of Italian Ministry of Health, 2006

Indicator: Yes/No

INRCA HEN Service results: Yes; the structural, technological and organizational characteristics of INRCA HEN center satisfy the accreditation standards defined by Italian Ministry of Health.

2. Structural indicator: Adequacy of places and methods of preserving enteral feeding tube formulas and devices at patient’s home

Why measure it? EN can be provided safely at home only if the patients and/or caregivers are well trained and if the environment at a patient’s home is safe. The ESPEN guidelines recommend that the environment for patients receiving HEN should be safe in order to administer EN without the risk of complications (R 58) and that hygiene standards should be established to prevent contamination of the home enteral product and to prevent HEN-related infections (R 59). However, ESPEN guidelines do not specify the control operating modes and standards [21]. Following INRCA protocol, on the occasion of each home visit, the home visiting staff checks out hygienic conditions and temperatures of sites at which enteral feeding tube formulas and devices are stored at a patient’s home. The results concerning the average number of correctly preserved feeding formulas and devices are shown in Table 1. Information provided by this indicator has to be used when interpreting the indicators of HEN-related complications, the outcomes of the therapy and when comparing the costs of different centers. This indicator has to be measured monthly in order to intervene immediately if the environment is not safe.

Standard: INRCA protocol

Indicator: Numerator: Number of correctly preserved and managed enteral feeding formulas and devices at home per month; Denominator: Total number of home visits per month.

3. Process indicator (organizational): Frequency of follow-up visits at patient’s home

Why measure it? HEN’s goal is to provide most of the services related to this specific therapy at home, without the need to resort to outpatient visits or hospitalizations. The efficacy of the transfer of the therapy at home and patient’s safety largely depends on follow-up home visits whose frequency and contents are not precisely defined by any guideline. According to IRCCS INRCA protocol, follow-up home visits for frail geriatric patients with comorbid conditions are performed on a monthly basis. The frequency of follow-up visits for geriatric patients followed by INRCA HEN center is shown in Table 2. Follow-up home visits are crucial for the prevention, recognition and prompt resolution of main complications of HEN (patient safety) and for the reduction of outpatient visits and hospitalizations (patient safety, patient Qol, patient costs, healthcare cost). Information provided by this indicator is useful to assess the patient’s safety and to interpret and to compare the frequency of HEN-related complications, the outcomes of the therapy (hospitalizations for complications of HEN) and costs between different HEN centers. The indicator may be measured annually.

Standard: INRCA protocol

Indicator: Numerator: Number of home visits performed per year; Denominator: Number of days of HEN therapy provided (net of hospitalizations) per year.

4. Process indicator (organizational): Frequency of outpatient visits for patients treated with HEN

Why measure it? Outpatient visits negatively affect safety and Qol of very old and frail patients whose transfer is difficult, and it frequently causes the worsening of their overall clinical conditions. Patients’ transfer in ambulance also represents an additional cost for patients and caregivers. Reduction of outpatient visits is an important objective of home services. At present, not all the measurements, assessments and interventions related to the management of HEN may be performed at patient’s home. According to INRCA protocol, the replacement of the NGT must be performed in the hospital in order to verify its correct positioning by X-rays and to ensure patient safety. This causes frequent visits to the emergency room or outpatient visits. Other services that sometimes have to be carried out in the clinic are dysphagia assessment and weight measurement in particularly complex patients or in those that do not have the necessary equipment at home. The frequency of outpatient visits of INRCA HEN patients is shown in Table 3. Information provided by this indicator is useful to assess the patient’s safety, Qol and to evaluate the overall costs of EN therapy. The indicator may be measured annually.

Standard: None. No guideline or protocol defines which assessments and interventions should be performed exclusively at home.

Indicator: Numerator: Number of patients treated with HEN who had at least 1 outpatient visit per year; Denominator: Number of patients treated with HEN yearly.

5. Outcome indicator: Frequency of complications of HEN therapy

Why measure it? HEN therapy may be associated with different complications: mechanical (tube-related), gastrointestinal and metabolic. The frequency of different complications of HEN therapy among INRCA HEN service is shown in Table 4. As recommended by the Italian Ministry of Health and in ESPEN guidelines (R 45), detecting benefits and harms of nutritional therapy and its outcomes is a prerequisite for safe, effective and ethically responsible care [20,21]. This indicator is used to assess patient safety and Qol. To assess patient safety and the efficacy of home service, this indicator has to be analyzed and interpreted together with indicators of frequency of outpatient visits, hospitalizations and deaths by HEN complications. It has to be measured annually.

Standards: None. No guideline or protocol defines a cut-off number of complications per patient for the definition of the efficacy and safety of a HEN service.

Indicator: Numerator: Number of complications of HEN therapy (tube related, metabolic, gastrointestinal and overall) per year; Denominator: Number of patients per year.

6. Outcome indicator: Frequency of hospitalizations for complications of HEN therapy

Why measure it? Old, frail patients treated with HEN are frequently hospitalized for reasons different from complications of HEN therapy. In order to assess the outcomes of HEN, it is important to collect precise data on causes of hospitalizations. This indicator shows how frequently complications attributable to HEN therapy have to be solved in hospital. Our results are shown in Table 5. It offers information on patient safety, Qol and on the effectiveness of home nutritional therapy in reducing the in-hospital treatments and costs. It has to be measured annually.

Standard: None. No guideline or protocol defines a cut-off for number of hospitalizations for complications of HEN for the definition of the efficacy and safety of a HEN service.

Indicator: Numerator: Number of hospitalizations for HEN related complications per year; Denominator: Total number of hospitalizations per year.

7. Outcome indicator: Length of hospital stays for complications of HEN therapy

Why measure it? In order to assess the cost effectiveness of home nutritional therapy, it is important to collect precise data on length of hospital stay for HEN related complications. It is also important when assessing the patients Qol and safety, given that the patient’s condition during hospitalization usually worsens, while healthcare costs increase. The results of IRCCS INRCA HEN center are shown in Table 6. This indicator has to be measured annually.

Standard: None. No guideline or protocol defines a cut-off of number of the days spent in the hospital for complications of HEN for the definition of the efficacy and safety of a HEN service and of its cost effectiveness.

Indicator: Numerator: Length of hospital stays for HEN related complications per year; Denominator: Total number of days of hospitalizations per year.

8. Outcome indicator: Frequency of deaths following hospitalizations for complications of HEN therapy

Why to measure it? In geriatric patients treated with HEN, death is mostly caused by complications related to underlying diseases. Only rarely do hospitalizations for complications of HEN therapy end with death. In order to assess the outcomes of HEN, it is important to collect precise data on causes of death. The results of IRCSS INRCA HEN service are shown in Table 7. This indicator provides information on how frequently complications attributable to HEN therapy led to death. This indicator offers important information on the safety and effectiveness of the therapy in patients with multiple diseases and it informs on outcomes of HEN therapy. It has to be measured annually. 

Standard: None. No guideline or protocol defines a cut-off for number of deaths following the hospitalization for complications of HEN therapy for the definition of the efficacy and safety of a HEN service. 

Indicator: Numerator: Number of deaths following the hospitalization for HEN therapy complications per year; Denominator: Total number of hospitalizations for HEN complications per year.

## 4. Discussion

Quality indicators are simple and effective tools for assessing the quality of healthcare. They may be used to track the performance of a health service over time, for comparing the results of different services and costs incurred to achieve them (benchmarking) and for the gap analyses, i.e., to measure how far the healthcare provided is from the standards defined for a specific disease and patient group. In 2015, we searched the literature for specific quality indicators and standards for HEN therapy. No indicators or studies assessing the safety of patients treated with HEN and the effectiveness of this home infusion therapy were found. After researching studies and documents containing instructions on how to define indicators, in the same year we developed eight (8) indicators, useful in evaluating the existence of the prerequisites for an efficient transfer of the therapy at home (structural indicators), the efficacy and safety of procedures adopted and to assess our results. 

We first tested these indicators in 2015. We have been tracking our activity regularly using them ever since. Considering that there are no similar data available in the literature, the characteristics of the indicators that we developed, in addition to our clinical results as measured by them, cannot be compared with other experiences and results of other centers. However, with reference to the characteristics of the indicators, we can affirm that all indicators presented in this work satisfy some important criteria that have to be observed when constructing indicators [36,37]. They are relevant, i.e., pertinent to objectives of nutritional therapy which has to be provided at patients’ homes (HEN). They are measurable and clear, i.e., there is no ambiguity about what is being measured or about how to interpret their results. Given that data needed to elaborate them can be easily obtained, the “practicality” criterion is also satisfied. Finally, they are reliable, i.e., they provide a basis for confident decision-making. 

Nevertheless, the elaboration of indicators is only the first step of the quality system management which is a multistage process. Although the indicators proposed in this manuscript may be—and actually are—used by our service for evidence-based planning and informed decision-making, in order to extend their use, they should be validated [38,39,40]. Normally, to develop indicators that have face and content validity, a systematic, guideline-driven approach of the RAND-modified Delphi-method in four steps is used. The first two steps of that method consist of a systematic review of the literature and the generation of indicators based on the literature review. The following steps, which are still to be accomplished, consist of the selection of expert panels, which should rate the preliminary indicators and then discuss and rate them again. Face validity should also be assessed. During that process experts could suggest additional quality indicators. In this regard, it must be said that many aspects related to the structure, process and outcome have not been covered in this work. The composition of the multidisciplinary team, the characteristics of the patient and/or caregiver training program and contents of the home visit, the achievement of objectives in terms of improvement of nutritional status and the assessment of patient and caregiver satisfaction are just some of them. It should also be specified that the elaboration and calculation of the indicators requires a large amount of very detailed and punctual data, which not all services may have. 

However, although the quality indicators presented in this manuscript have been developed specifically for the needs of our HEN service for geriatric patients—using, in some cases, our internal protocols as standards—the evaluation of other services with the same indicators is possible and would actually allow for a first comparison between their results and reference standards. 

## 5. Conclusions

Monitoring the quality of HEN services should become a priority issue. Quality indicators are a simple tool that might be used for this purpose. To measure efficacy, safety and patient centeredness of HEN services, specific quality indicators should be used. We have made a first attempt and developed eight indicators for geriatric HEN service. Indicators proposed should be discussed and validated by the scientific community and numerous other indicators should be developed.

## Figures and Tables

**Table 1:** Average number of correctly preserved enteral feeding formulas and devices at home; INRCA HEN Service (2015–2021).

	2015	2016	2017	2018	2019	2020	2021
Average n. of correctly preserved and managed enteral feeding tube formulas and devices at home (%)	99.1	98.8	99.7	98	99.6	N.A. *	99.5

* N.A. not available (home visits were not performed due to COVID-19 pandemic).

**Table 2:** Frequency of follow up home visits; INRCA HEN Service (2015–2021).

	2015	2016	2017	2018	2019	2020	2021
Frequency of follow-up visits (days)	29.13	29.18	29.07	29.8	29.8	N.A.*	29.6

* N.A. not available (home visits were not performed due to COVID-19 pandemic).

**Table 3:** Frequency of outpatient visits for patients treated with HEN; INRCA HEN Service (2015–2021).

	2015	2016	2017	2018	2019	2020	2021
Frequency of outpatient visits	39%	32%	36%	31%	34%	N.A.*	44%

* N.A. not available (home visits were not performed due to COVID-19 pandemic).

**Table 4:** Frequency of complications of HEN therapy; INRCA HEN Service (2015–2021).

	2015	2016	2017	2018	2019	2020	2021
Tube related complications/pt	2.4	2.5	2.3	2.2	2.5	2.9	2.7
Gastrointestinal complications/pt	1.6	1.0	1.1	0.6	0.5	0.6	1.0
Metabolic complications/pt	0.2	0.4	0.5	0.2	0.2	0.2	0.2
Total complications	4.2	3.9	3.9	3.0	3.2	3.8	3.9

**Table 5:** Frequency of hospitalizations for complications of HEN therapy (% of total hospitalizations), INRCA HEN service (2015–2021).

	2015	2016	2017	2018	2019	2020	2021
Hospitalizations for complications of HEN therapy as % of total hospitalizations of patients treated with HEN	16%	17%	13%	13%	21%	15%	11%

**Table 6:** Length of hospital stay for complications of HEN therapy (% of total LOS), INRCA HEN service (2015–2021).

	2015	2016	2017	2018	2019	2020	2021
Days spent in hospital for complications of HEN therapy as % of total days of hospitalizations of patients treated with HEN	10%	18%	15%	7%	14%	10%	8%

**Table 7:** Frequency of deaths following hospitalizations for complications of HEN therapy; INRCA HEN service (2015–2021).

	2015	2016	2017	2018	2019	2020	2021
Deaths following hospitalizations for complications of HEN therapy as % of total deaths	13%	8%	4%	14%	10%	11%	11%

## Data Availability

Data are available upon reasonable and motivated request to be presented to the authors.
